# Functional Assessment of the Foot Undergoing Percutaneous Achilles Tenotomy in Term of Gait Analysis

**DOI:** 10.1155/2016/1973403

**Published:** 2016-08-29

**Authors:** Yu-Bin Liu, Shu-Yun Jiang, Li Zhao, Yan Yu, Xu-Chen Tao, Da-Hang Zhao

**Affiliations:** ^1^Department of Pediatric Orthopaedics, Xin-Hua Hospital, Shanghai Jiao Tong University School of Medicine, Shanghai 200092, China; ^2^Gait Lab, Yueyang Hospital, Shanghai University of Traditional Chinese Medicine, Shanghai 200437, China

## Abstract

*Background.* This study was designed to evaluate the function of the foot undergoing the procedure of percutaneous Achilles tenotomy (PAT) in case of clubfoot management in terms of gait analysis.* Methods.* Nineteen patients with unilateral clubfeet were retrospectively reviewed from our database from July 2012 to June 2016. The result in all the cases was rated as excellent according to the scale of International Clubfoot Study Group (ICSG). The affected sides were taken as Group CF and the contralateral sides as Group CL. Three-dimensional gait analysis was applied for the functional evaluation of the involved foot.* Results.* Statistical difference was found in physical parameters of passive ankle dorsiflexion and plantar-flexion. No statistical difference was found in temporal-spatial parameters. There was statistical difference in kinematic parameters of total ankle rotation, ankle range of motion, and internal foot progression angle and in kinetic parameters of peak ankle power. No statistical difference was found in other kinematic and kinetic parameters.* Conclusions.* It is demonstrated that the procedure of PAT is safe and efficient for correcting the equinus deformity in case of clubfoot management and preserving the main function of Achilles tendon at the minimum of four-year follow-up.

## 1. Introduction

The Achilles tendon, that is, heel cord, is a tendon of the back of the leg and the strongest and thickest tendon in the human body. It serves as the function of connecting the soleus and gastrocnemius muscles to the os calcis to allow plantar flexion of the foot at the ankle and provides elastic energy storage in hopping, walking, and running [1]. The Achilles tendon is the most frequently ruptured tendon in the human body [2] with a higher incidence in males than females [3]. It is recommended that the intervention of Achilles tendon rupture should be undertaken soon, whether surgical or conservative treatment, to obviate late disability, pain, and healthcare cost [4]. However, it is still controversial whether operative or nonoperative procedure should be undertaken in the management of Achilles tendon rupture [5, 6].

Percutaneous Achilles tenotomy (PAT) is an important procedure in the clubfoot management using Ponseti method [7–9]. As reported, over 90% of cases required this procedure for correcting the equinus deformity [7, 8]. However, the debates still remain as to the healing and efficacy of Achilles tenotomy in the professional community of orthopaedists because of the established opinion that the orthopaedic intervention of the ruptured Achilles tendon should be undertaken soon. The evidence of the healing of Achilles tendon has been obtained from the ultrasonographic and MRI studies [10, 11]. However, it was considered in the previous studies that the Achilles tendon, undergoing the rupture and healing of Achilles tendon, would never be as strong as the original one because it was supposed that the gap of tendon rupture was filled with fibrous tissues even after healing [12]. It was revealed that the rerupture rate with conservative treatment was from 12% [13] to 13.4% [14]. As to the functional evaluation of Achilles tendon, clinical questionnaire consisting of the items as pain, functional activity level assessments, and muscle strength testing was established to assess the recovery of injured tendons in vivo [15]. In clinical practice, two typical tests, including Thompson sign and heel raise test, were conducted to evaluate the Achilles tendon function. The outcome measurement of unilateral clubfoot treated with the Ponseti method including the PAT procedure with a particular reference to the data of gait analysis has been poorly reported in the literature. Percutaneous Achilles tenotomy has been the critical procedure over the last decades in the management of congenital clubfoot. It is assumed that this procedure is taken as the model of Achilles tendon healing in a child. This study was designed to evaluate the function of the foot in which the Achilles tenotomy was performed for correcting the equinus deformity in case of clubfoot management in terms of clinical assessment and gait analysis.

## 2. Materials and Methods

The medical records were retrospectively reviewed from our database to identify patients with idiopathic clubfeet treated with Ponseti method during the period between July 2012 and June 2016. All the clubfoot cases were treated by a single orthopedist. The patients, who had the unilateral clubfoot, had undergone the procedure of PAT, and had finished the recommended bracing phase and the evaluation of gait analysis, were included for the present study. The exclusion criteria were as follows: (1) the postural, syndromic, and neurological clubfeet, (2) the cases still remaining in the course of treatment without the evaluation using gait analysis, and (3) the cases, which underwent the surgical treatments such as the procedures of transfer of anterior tibialis tendon (TATT), Ilizarov technique, and/or extensive soft tissue release. Informed consent was obtained from all the parents. This study was approved by the institutional ethics committee.

The result in all the cases (16 boys and 3 girls) was rated as excellent according to the Functional Rating Scales of International Clubfoot Study Group (ICSG). There were 14 clubfeet on the right side and 5 clubfeet on the left side. The mean age was 4.5 years (range, 4 to 6 years) at the time when the test of gait analysis was undertaken, with mean body height of 108.6 cm (range, 103 to 122 cm) and mean body weight of 18.9 kg (range, 16 to 23.5 kg). Fourteen patients firstly came to visit us at our clinic with the mean presentation age of 82.9 days (range, 11 days to 7 months) while five patients received the previous treatments (mainly casting and PAT procedure) before referral to our clinic with the mean presentation age of 26 months (range, 8 months to 3 years). The mean Pirani score was 4.2 points (3 to 6 points) in the 15 feet from fifteen patients with age younger than 1 year while the remaining four feet from 4 patients with age older than 1 year were classified as moderate according to Dimeglio classification [16]. The initial complete correction was obtained in all the cases (100%), requiring mean numbers of 4.2 (2 to 6) casts before PAT procedure was performed. The affected sides of treated patients with clubfoot were grouped as Group CF while the contralateral normal sides were grouped as Group CL. A foot abduction orthosis (FAO) brace, which consisted of a pair of straight high-top shoes and a connected bar, was prescribed to prevent the relapse of deformity. The brace protocol was full-time use for the first 3 months and then 16 to 18 hours until the patient was 2 years old and then 14 to 16 hours until the patient was 4 years old. All the patients were followed up at the minimum of 4.5 years old.

All the subjects, who had finished the whole course of clubfoot management using Ponseti method, were regularly recommended to undertake the test of gait analysis for the functional evaluation in our partner institute-gait lab at Shanghai Yueyang Hospital. Anthropometric data, including height, weight, passive ankle dorsiflexion angle with knee extension, passive ankle plantar flexion angle, and thigh foot angle, were documented. Twenty-two passively reflective markers were placed on the standardized and specific anatomical landmarks. After the retroreflective markers were applied to the patients, they were instructed to walk barefoot at a self-selected speed over a 10 m walkway while the data capture was undertaken. Three-dimensional gait data was collected with the motion analysis system (Motion Analysis Corporation, USA). Four force plates (Advanced Mechanical Technology, Inc., Watertown, MA) were used for kinetic analysis. The spatial locations of the individual markers were recorded using twelve cameras (60 Hz) as the subjects walked. Temporal-spatial parameters, kinematic parameters, and kinetic parameters were collected as the gait summary measures for analysis. Calibration of the motion analysis system was performed each time before the subjects were taken for the test.

### 2.1. Statistical Analysis

Statistical analysis was performed using the statistical package of SPSS 17.0. Comparisons of groups in terms of clinical and physical evaluation, temporal-spatial parameters, and kinematic and kinetic variables were performed using paired* t*-test. Statistical significance was set at *p* < 0.05.

## 3. Results

### 3.1. Physical Examination

Anthropometrical characteristics of these two groups were presented in Table 1. Passive ankle dorsiflexion angle with knee extension in Group CF (15.20 ± 9.68°) was smaller than that in Group CL (26.40 ± 9.19°) with the statistically significant difference (*p* = 0.000). Significant difference was also statistically found in the parameter of passive ankle plantar flexion angle between these two groups (42.13 ± 7.28° for Group CF and 47.93 ± 6.70° for Group CL, *p* = 0.000). There was no significant difference in the physical parameter of thigh foot angle (9.53 ± 9.83° for Group CF and 10.07 ± 10.61° for Group CL, *p* = 0.752).

### 3.2. Temporal-Spatial Parameters

Table 2 shows the comparison of temporal-spatial parameters between Group CF and Group CL. The parameter of stride length in Group CF (73.10 ± 11.58 cm) was a little smaller than that in Group CL (73.59 ± 11.42 cm); however, no significant difference was found between these two groups (*p* = 0.092). There was no significant difference in the parameter of forward velocity between these two groups (79.72 ± 20.27 cm/s for Group CF and 79.93 ± 20.33 cm/s for Group CL, *p* = 0.446). The parameter of cadence in Group CF (129.96 ± 16.65 steps/min) was nearly identical to that in Group CL (129.98 ± 16.37 steps/min, *p* = 0.940). No significant difference was found in the parameter of single support time (%) between these two groups (37.56 ± 3.41 for Group CF and 38.16 ± 3.45 for Group CL, *p* = 0.159).

### 3.3. Kinematic Parameters

**Figure 1:**
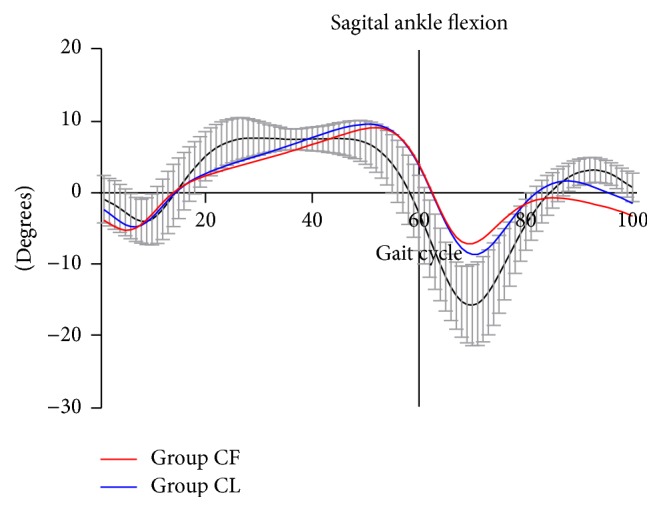
Averaged ankle range of motion in sagittal plane for Group CF (red) and Group CL (blue) over one complete gait cycle was compared. Positive values represent dorsiflexion, and negative values represent plantar flexion. Decreased plantar flexion at initial swing phase (toe off phase) was found for both Group CF and Group CL.

Table 3 shows the comparison of kinematic parameters between Group CF and Group CL. The total ankle rotation angle in coronal plane in Group CF (19.91 ± 5.10°) was significantly greater than that in Group CL (16.87 ± 4.91°, *p* = 0.011). There was no significant difference in the parameters of peak ankle dorsiflexion and peak ankle plantar flexion (*p* > 0.05, Figure 1). However, the parameter of total ankle range of motion was significantly greater in Group CL (21.95 ± 5.58°) than that in Group CF (19.20 ± 4.53°) (*p* = 0.022). Significant difference was also found in the parameter of internal foot progression angle (−1.38 ± 6.64° for Group CF and −8.09 ± 6.09° for Group CL, *p* = 0.002).

### 3.4. Kinetic Parameters

**Figure 2:**
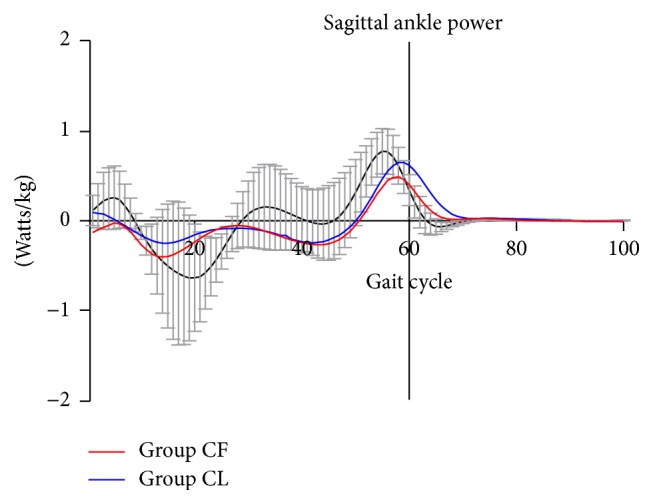
Averaged ankle power in sagittal plane for Group CF (red) and Group CL (blue) over one complete gait cycle was compared. Positive values are power generation (Gen) and negative values are power absorption (Abs). Ankle power at push-off phase was significantly decreased for Group CF in comparison with that for Group CL.

Table 4 shows the comparison of kinetic parameters between Group CF and Group CL. There was no significant difference in the parameters of peak ankle plantar flex moment between these two groups (0.61 ± 0.27 Nm/kg for Group CF and 0.71 ± 0.13 Nm/kg for Group CL, *p* = 0.197). No significant difference was found in the parameters of peak vertical ground reaction force (GRF) (1.03 ± 0.12 N/kg for Group CF and 1.06 ± 0.12 N/kg for Group CL, *p* = 0.265) and peak frontal propulsion force (0.15 ± 0.07 N/kg for Group CF and 0.15 ± 0.04 N/kg for Group CL, *p* = 0.882) between these two groups either. Significant difference was found in the parameter of peak ankle power (0.55 ± 0.23 Watts/kg for Group CF and 0.91 ± 0.47 Watts/kg for Group CL, *p* = 0.001) between these two groups (Figure 2).

## 4. Discussion

Gait analysis has been used as a useful tool for assessing the functional outcomes by quantitatively measuring the motion of walking in terms of body movements, body mechanics, and the activity of the muscles to evaluate treatment outcome of clubfeet functionally and objectively over the last decades [17–20]. It is controversial as to the issue whether the Achilles tendon should be completely transected. As it was warned, the tendon continuity must be maintained with percutaneous Achilles tendon lengthening rather than Achilles tenotomy [21, 22] fearing the spastic gastrocnemius-soleus muscle contraction of the proximal tendon end, which is supposed to prevent the tendon-to-tendon healing and lead to myotendinous incompetence. However, Berg [23] reported that inadvertent Achilles tenotomy could rebuild the tendon continuity as long as the patients (3 children and 2 adults) were immobilized for a minimum of 2 months in a short leg walking cast. From the viewpoint of pediatric orthopedists in their practice of clubfoot management, the procedure of PAT has since been a safe and effective procedure to correct the equinus deformity once the forefoot is adequately abducted [24]. As to the issue of heel cord healing, Agarwal et al. [10] demonstrated that functional continuity of the Achilles tendons could be rebuilt at 4 weeks' time after tenotomy in terms of clinical and ultrasonographic measurements. Mangat et al. [25] reported that the complete healing of transected Achilles tendons required the time of at least twelve weeks although there was evidence of continuity of the Achilles tendon at three weeks after tenotomy. Saini et al. [11] demonstrated that the continuity of the Achilles tendon in all cases could be reestablished at the end of 6 weeks in terms of clinical assessment and 6 months of the tenotomy in terms of MRI imaging evaluation. All the data from ultrasonographic and MRI studies supported that the Achilles tendon did regenerate following the procedure of PAT. In our clinical practice, the healing of Achilles tendon was recognized from the clinical evaluations including heel raise test and walking gait based on the general observation. However, the specific difference of foot function between the feet with and without the procedure of PAT procedure has been poorly reported using the detailed methods of gait analysis. In the present study, we aimed to quantify the foot function of unilateral clubfoot, which underwent the procedure of PAT in comparison with the contralateral foot in terms of gait analysis.

It is suggested based on the findings of the present study that the majority of the affected feet have obtained the satisfactory gait and function following the procedure of PAT in terms of the temporal-spatial parameters during the complete gait cycle. The values of passive ankle dorsiflexion and plantar flexion angles presented the significant reduction angles for Group CF in comparison with those for Group CL (*p* < 0.05). However, the angles of ankle passive dorsiflexion and plantar flexion for Group CF were great enough to complete the whole gait cycle without any limitation (Table 3, *p* > 0.05). Dynamic ankle range of motion for Group CF was found to present the dramatic reduction (Table 3, *p* < 0.05). Mindler et al. [26] also reported significantly decreased range of motion at the ankles in the Ponseti group in comparison with that in the controls. This may be attributed to the underlying musculoskeletal pathologies as well as the long time casting immobilization. The mean foot progression angle (−1.38 ± 6.64°) for Group CF also presented the significant reduction in comparison with that for Group CL (−8.09 ± 6.09°). However, it was more externally oriented comparing with that reported by Karol et al. [19] and more internally oriented comparing with that reported by Mindler et al. [26]. We have found that the subjects of Group CF walked similarly to those of Group CL in terms of temporal-spatial parameters such as stride length, forward velocity, cadence, and single support time (*p* > 0.05). The similar results were reported in previous studies [27–29]. Aksahin et al. [30] found that clubfoot patients, who underwent posteromedial release surgery, walked slower than those from the control group with short steps. The kinetic parameters were comparable between these two groups except for the parameter of peak ankle power. The mean value of the peak ankle power in the Group CF undergoing the procedure of PAT (0.55 Watts/kg) was 60.4% of that in Group CL (0.91 Watts/kg). Reduced ankle power percentages relative to controls have been reported in some other studies [19, 26, 31]. In the present study, peak ankle powers of Group CF (0.55 Watts/kg) and Group CL (0.91 Watts/kg) presented the great reduction comparing with those in the previous studies (2.2 Watts/kg for Ponseti group and 2.6 Watts/kg for control group [26]; 1.19 watts/kg for surgical group and 1.25 for control group [30]) as to the outcome evaluation of clubfoot treatment. This may be attributed supposedly to the variations in regions, races, and demographic characteristics among the different reports. Ankle power is affected by both range of motion and muscle strength [19]. Because normal ankle plantar flexion is requisite for foot push-off, insufficient ankle plantar flexion may be an important factor for difficulty of foot propulsion. In the present study, peak ankle plantar flexion along with ankle plantar flex moment was comparable between Group CF and Group CL (*p* > 0.05). The reduced ankle power for Group CF may be supposedly attributed to the procedure of PAT. However, it was difficult to draw the conclusion because the smaller gastrosoleus muscle in the bulk of the most clubfeet patients may lead to relative weakness and reduced ankle power. Previous studies also supported an association between clubfoot and diminished ankle power [29, 32, 33]. No significant difference was found in other kinetic parameters such as peak ankle plantar flex moment, peak vertical ground reaction force (GRF), and peak frontal propulsion force.

There were a few limitations in the present study. Assuming that the foot could be taken as a single rigid segment, pivoting at the ankle joint, the traditional single-segment foot model (Helen Hayes model) was used for gait analysis in the present study. However, multisegment foot model may enhance the accuracy of the gait analysis data and provide valuable additional information among the different foot segments including Milwaukee Foot Model, Oxford Foot Model, and Leardini (IOR) Foot Model [34]. Either way it is not unusual that the child does not walk in their “normal” fashion while he or she is undergoing the test of gait analysis. Finally, the threshold for what is considered as pathologic versus functional gait in children is still controversial. However, we applied paired *t*-test for statistical analysis and observed the gait difference in the children with unilateral clubfoot undergoing the procedure of PAT in comparison with that in contralateral normal foot. This may be supposed to reduce the bias among the groups.

As revealed in this study, the feet following the procedure of PAT showed no obvious gait deviation in outcome measure with the reference to the temporal-spatial, kinematic, and kinetic parameters of gait analysis. Based on the findings from this study, it is demonstrated that good or excellent results can be achieved in the foot undergoing the procedure of PAT at the minimum of four-year follow-up. The Achilles tendon healed well in terms of clinical assessment and gait analysis. It is recommended that the procedure of PAT be safe and efficient for correcting the equinus deformity in case of clubfoot management and preserving the main function of Achilles tendon.

## Figures and Tables

**Table 1:** Physical parameters.

Parameters	Group CF	Group CL	*t* value	*p* value
Passive ankle dorsiflexion angle (deg)	15.20 ± 9.68	26.40 ± 9.19	*t* = −4.836	*p* = 0.000
Passive ankle plantar flexion angle (deg)	42.13 ± 7.28	47.93 ± 6.70	*t* = −5.908	*p* = 0.000
Thigh foot angle (deg)	9.53 ± 9.83	10.07 ± 10.61	*t* = −0.323	*p* = 0.752

**Table 2:** Temporal-spatial parameters.

Parameters	Group CF	Group CL	*t* value	*p* value
Stride length (cm)	73.10 ± 11.58	73.59 ± 11.42	*t* = −1.777	*p* = 0.092
Forward velocity (cm/s)	79.72 ± 20.27	79.93 ± 20.33	*t* = −0.779	*p* = 0.446
Cadence (steps/min)	129.96 ± 16.65	129.98 ± 16.37	*t* = −0.076	*p* = 0.940
Single support time (%)	37.56 ± 3.41	38.16 ± 3.45	*t* = −1.470	*p* = 0.159

**Table 3:** Kinematic parameters.

Parameters	Group CF	Group CL	*t* value	*p* value
Total ankle rotation (deg)	19.91 ± 5.10	16.87 ± 4.91	*t* = 2.879	*p* = 0.011
Peak ankle dorsiflexion (deg)	9.99 ± 4.90	10.85 ± 4.82	*t* = −0.832	*p* = 0.416
Peak ankle plantar flexion (deg)	−9.21 ± 6.57	−11.10 ± 6.84	*t* = 1.565	*p* = 0.135
Ankle range of motion (deg)	19.20 ± 4.53	21.95 ± 5.58	*t* = −2.509	*p* = 0.022
Internal foot progression angle (deg)	−1.38 ± 6.64	−8.09 ± 6.09	*t* = 3.613	*p* = 0.002

**Table 4:** Kinetic parameters.

Parameters	Group CF	Group CL	*t* value	*p* value
Peak ankle plantar flex moment (Nm/kg)	0.61 ± 0.27	0.71 ± 0.13	*t* = −1.352	*p* = 0.197
Peak vertical GRF (N/kg)	1.03 ± 0.12	1.06 ± 0.12	*t* = −1.160	*p* = 0.265
Peak frontal propulsion (N/kg)	0.15 ± 0.07	0.15 ± 0.04	*t* = −0.151	*p* = 0.882
Peak ankle power (Watts/kg)	0.55 ± 0.23	0.91 ± 0.47	*t* = −3.982	*p* = 0.001

GRF: ground reaction force.

## References

[1] Lichtwark G. A., Wilson A. M. (2005). *In vivo* mechanical properties of the human Achilles tendon during one-legged hopping. *The Journal of Experimental Biology*.

[2] Maffulli N., Waterston S. W., Squair J., Reaper J., Douglas A. S. (1999). Changing incidence of Achilles tendon rupture in Scotland: a 15-year study. *Clinical Journal of Sport Medicine*.

[3] Möller A., Åström M., Westlin N. E. (1996). Increasing incidence of Achilles tendon rupture. *Acta Orthopaedica Scandinavica*.

[4] Thomopoulos S., Parks W. C., Rifkin D. B., Derwin K. A. (2015). Mechanisms of tendon injury and repair. *Journal of Orthopaedic Research*.

[5] Gulati V., Jaggard M., Al-Nammari S. S. (2015). Management of achilles tendon injury: a current concepts systematic review. *World Journal of Orthopaedics*.

[6] van der Eng D. M., Schepers T., Goslings J. C., Schep N. W. L. (2013). Rerupture rate after early weightbearing in operative versus conservative treatment of Achilles tendon ruptures: a meta-analysis. *The Journal of Foot and Ankle Surgery*.

[7] Zhao D., Li H., Zhao L., Liu J., Wu Z., Jin F. (2014). Results of clubfoot management using the Ponseti method: do the details matter? A systematic review. *Clinical Orthopaedics and Related Research*.

[8] Morcuende J. A., Dolan L. A., Dietz F. R., Ponseti I. V. (2004). Radical reduction in the rate of extensive corrective surgery for clubfoot using the Ponseti method. *Pediatrics*.

[9] Dobbs M. B., Rudzki J. R., Purcell D. B., Walton T., Porter K. R., Gurnett C. A. (2004). Factors predictive of outcome after use of the Ponseti method for the treatment of idiopathic clubfeet. *The Journal of Bone & Joint Surgery—American Volume*.

[10] Agarwal A., Qureshi N. A., Kumar P., Garg A., Gupta N. (2012). Ultrasonographic evaluation of Achilles tendons in clubfeet before and after percutaneous tenotomy. *Journal of Orthopaedic Surgery*.

[11] Saini R., Dhillon M. S., Tripathy S. K. (2010). Regeneration of the Achilles tendon after percutaneous tenotomy in infants: a clinical and MRI study. *Journal of Pediatric Orthopaedics B*.

[12] Webb J. M., Bannister G. C. (1999). Percutaneous repair of the ruptured tendo Achillis. *The Journal of Bone & Joint Surgery—British Volume*.

[13] Lo I. K. Y., Kirkley A., Nonweiler B., Kumbhare D. A. (1997). Operative versus nonoperative treatment of acute achilles tendon ruptures: a quantitative review. *Clinical Journal of Sport Medicine*.

[14] Cetti R., Christensen S.-E., Ejsted R., Jensen N. M., Jorgensen U. (1993). Operative versus nonoperative treatment of Achilles tendon rupture. A prospective randomized study and review of the literature. *The American Journal of Sports Medicine*.

[15] Zhang L. N., Wan W. B., Wang Y. X. (2016). Evaluation of elastic stiffness in healing achilles tendon after surgical repair of a tendon rupture using in vivo ultrasound shear wave elastography. *Medical Science Monitor*.

[16] Dimeglio A., Bensahel H., Souchet P., Mazeau P., Bonnet F. (1995). Classification of clubfoot. *Journal of Pediatric Orthopedics Part B*.

[17] Jeans K. A., Erdman A. L., Jo C. H., Karol L. A. (2016). A longitudinal review of gait following treatment for idiopathic clubfoot: gait analysis at 2 and 5 years of age. *Journal of Pediatric Orthopaedics*.

[18] Gottschalk H. P., Karol L. A., Jeans K. A. (2010). Gait analysis of children treated for moderate clubfoot with physical therapy versus the Ponseti cast technique. *Journal of Pediatric Orthopaedics*.

[19] Karol L. A., Jeans K., ElHawary R. (2009). Gait analysis after initial nonoperative treatment for clubfeet: intermediate term followup at age 5. *Clinical Orthopaedics and Related Research*.

[20] El-Hawary R., Karol L. A., Jeans K. A., Richards B. S. (2008). Gait analysis of children treated for clubfoot with physical therapy or the Ponseti cast technique. *Journal of Bone and Joint Surgery A*.

[21] Moreau M. J., Lake D. M. (1987). Outpatient percutaneous heel cord lengthening in children. *Journal of Pediatric Orthopaedics*.

[22] Hatt R. N., Lamphier T. A. (1947). Triple hemisection: a simplified procedure for lengthening the Achilles tendon. *The New England Journal of Medicine*.

[23] Berg E. E. (1992). Percutaneous achilles tendon lengthening complicated by inadvertent tenotomy. *Journal of Pediatric Orthopaedics*.

[24] Alam M. T., Akber E. B., Alam Q. S. (2015). Outcome of percutaneous tenotomy in the management of congenital talipes equino varus by Ponseti method. *Mymensingh Medical Journal*.

[25] Mangat K. S., Kanwar R., Johnson K., Korah G., Prem H. (2010). Ultrasonographic phases in gap healing following Ponseti-type Achilles tenotomy. *The Journal of Bone & Joint Surgery—American Volume*.

[26] Mindler G. T., Kranzl A., Lipkowski C. A. M., Ganger R., Radler C. (2014). Results of gait analysis including the oxford foot model in children with clubfoot treated with the ponseti method. *The Journal of Bone & Joint Surgery—American Volume*.

[27] Karol L. A., Concha M. C., Johnston C. E. (1997). Gait analysis and muscle strength in children with surgically treated clubfeet. *Journal of Pediatric Orthopaedics*.

[28] Hee H. T., Lee E. H., Lee G. S. (2001). Gait and pedobarographic patterns of surgically treated clubfeet. *The Journal of Foot and Ankle Surgery*.

[29] Davies T. C., Kiefer G., Zernicke R. F. (2001). Kinematics and kinetics of the hip, knee, and ankle of children with clubfoot after posteromedial release. *Journal of Pediatric Orthopaedics*.

[30] Aksahin E., Yuksel H. Y., Yavuzer G., Muratli H. H., Celebi L., Bicimlioglu A. (2010). Quantitative gait characteristics of children who had successful unilateral clubfoot operation. *Acta Orthopaedica et Traumatologica Turcica*.

[31] Duffy C. M., Salazar J. J., Humphreys L., McDowell B. C. (2013). Surgical versus ponseti approach for the management of CTEV: a comparative study. *Journal of Pediatric Orthopaedics*.

[32] Aronson J., Puskarich C. L. (1990). Deformity and disability from treated clubfoot. *Journal of Pediatric Orthopaedics*.

[33] Theologis T. N., Harrington M. E., Thompson N., Benson M. K. D. (2003). Dynamic foot movement in children treated for congenital talipes equinovarus. *Journal of Bone and Joint Surgery B*.

[34] Novak A. C., Mayich D. J., Perry S. D., Daniels T. R., Brodsky J. W. (2014). Gait analysis for foot and ankle surgeons—topical review, part 2: approaches to multisegment modeling of the foot. *Foot and Ankle International*.

